# Nitride fuel for Gen IV nuclear power systems

**DOI:** 10.1007/s10967-018-6316-0

**Published:** 2018-11-10

**Authors:** Christian Ekberg, Diogo Ribeiro Costa, Marcus Hedberg, Mikael Jolkkonen

**Affiliations:** 1 Nuclear Chemistry/Industrial Materials Recycling, Chalmers, Göteborg, Sweden; 2 Division of Nuclear Engineering, Kungliga Tekniska Högskolan, Stockholm, Sweden; 3 Westinghouse Electric Sweden AB, Västerås, Sweden

**Keywords:** Nuclear fuel, Nitride nuclear fuels, Gen IV, Production of nitrides, Nuclear fuel recycling, Dissolution of nitrides

## Abstract

Nuclear energy has been a part of the energy mix in many countries for decades. Today in principle all power producing reactors use the same techniqe. Either PWR or BWR fuelled with oxide fuels. This choice of fuel is not self evident and today there are suggestions to change to fuels which may be safer and more economical and also used in e.g. Gen IV nuclear power systems. One such fuel type is the nitrides. The nitrides have a better thermal conductivity than the oxides and a similar melting point and are thus have larger safety margins to melting during operation. In addition they are between 30 and 40% more dense with respect to fissile material. Drawbacks include instability with respect to water and a sometimes complicated fabrication route. The former is not really an issue with Gen IV systems but for use in the present fleet. In this paper we discuss both production and recycling potential of nitride fuels.

## Introduction

Nuclear power is today a disputed technique although it is in principle CO_2_ free and highly energetic. However, the current nuclear systems are rather inefficient, only about 1% of the inherent energy is used, and the waste need to be stored for about 100,000 years before its radiotoxicity is similar to the originally mined uranium. In some countries such as France there is a reprocessing system used where the plutonium is used in the refabricating of mixed oxide fuel that is once more used in a power producing reactor. This is, however, only done once increasing the energy utilisation to about 1.2% but saving about 17% of freshly mined uranium [1] In the last 2 decades a complete circular use of the fuel material, the so called Gen IV nuclear power systems have emerged as a potential solution to the aforementioned issues. The following goals for Gen IV were defined previously in the Generation IV International Forum (GIF) [2, 3]: sustainability, safety and reliability, economic competitiveness, and proliferation resistance and physical protection. Such a system comprise fast reactors, separation facilities for recovery of the still useful actinides and a fuel fabrication plant to complete the recycling. Naturally, several questions arise when discussing a new power production systems such as which combination of coolant, cladding and fuel to use in the reactor as well as a selection of separation system and fuel type and fabrication route.

Over the years there has been different investigations to find the optimal nuclear fuel. Initially, in the 1940s, uranium metal was chosen as a potential candidate mainly because its high thermal conductivity and fissile density. However, its low melting point, associated with its phase transformation during heating, chemical instability, and high fission gas swelling, became a drawback to its industrial application. To work around these problems, the most promising alternative was the ceramic oxide fuel, specifically the uranium dioxide (UO_2_), which has high melting point, good chemical stability and compatibility with the fission products, and reasonable thermal stability during burn-up. The main downside, otherwise, was its very low thermal conductivity.

Parallel to uranium dioxide development, many research projects have been carried out in order to consider other fuel options, such as nitrides, carbides and silicides [4]. Among them, nitrides has shown many superior qualities over the standard oxide fuel such as:

- Higher fissile density (40% more uranium in UN than in UO_2_) [4]: leading to higher conversion ratios, and potentially higher burn-ups.
- *Higher thermal conductivity* reduction of the fuel centreline temperature, decrease in the energy stored per unit mass while increasing the margin for fuel melting [5, 6], and delay the migration of fission products and actinides, which positively affects the fuel swelling [7].
- *Reprocessing* good dissolution in nitric acid (HNO_3_), making this fuel compatible with the PUREX process, which uses HNO_3_ for dissolution of spent fuel [8].
- *Stability* good chemical compatibility with most potential cladding materials, as well as irradiation stability [4].
- *Longer fuel cycle time* owing to neutronic behaviour of UN in the core [9], the fuel cycle could increase from the commonly applied 18 months (standard UO_2_) to about 25 months, based on a burnup of 50 GWd/tU. This increase leads to fewer shut downs for reloading, thus being an economical benefit for nitride fuel implementation [4].

On the other hand, there are some drawbacks regarding the nitride fuels:

- The production of minor actinide (or even plutonium) containing nitride fuel is not straight forward and require some difficult production steps.
- Oxidation resistance: the nitride pellets readily oxidizes in superheated steam, with complete degradation obtained within 1 h in 0.50 bar steam at 500 °C for UN [10]. The as-manufactured nitride powder is pyrophoric, which means that it reacts immediately with oxygen from air, for instance. Thus, the atmosphere during handling the UN powder must be oxygen-free, which requires additional implementation for industrial scale production [11].
- Fuel cost: mainly due to the fact that the nitrogen component has to be highly enriched in ^15^N to increase the neutron economy and avoid the (n, p) formation of ^14^C from ^14^N [12]. This reaction also enhances the amount of radioactive material in the spent fuel, which is undesirable. However, the additional costs related to nitrogen enrichment are offset by lower uranium enrichment requirements, as well as the reduced number of fuel assemblies in each fuel reload, resulting in an estimated US $5 million per year savings [4].

**Fig. 1:**
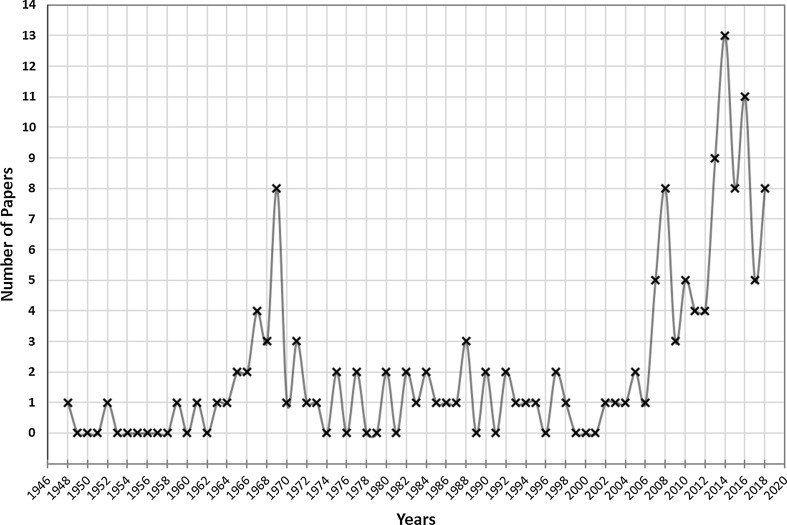
Number of uranium nitride papers published though the years. The search was done using Scopus website, with “uranium nitride” as part of the paper title; others nitrides-type were not counted [15]

Since the earliest studies, back in the 1900s, many methods of obtaining the nitrides have been developed, such as: direct nitriding, hydriding–dehydriding–nitriding, carbothermic nitriding, oxidative ammonolysis, and others [13, 14]. An overview of uranium nitride papers published through the years, from 1948 to 2018 is shown in Fig. 1 [15].

There are two regions in which the number of papers increased considerably. The first, between 1964 and 1970, might be related to the most comprehensive uranium nitride characterization as well as the new preparation routes from uranium tetrachloride and tetrafluoride, part of the (here mentioned) “golden age” regarding uranium nitride research and development [16–18]. The second, most clearly defined after 2011, is responsible for around 42% of the published papers, as seen above. This corresponds to something about 9 papers per year, against approximately 1 paper per year from 1946 to 2010. That behaviour is mainly impacted by the response from the scientific community after the Fukushima Daiichi accident on 11 March 2011, caused by the earthquake and the following tsunami. This scenario exceeds the severity of the design basis accidents (DBA) [19]. Afterwards, the international goal was changed from just improving the existing UO_2_-zirconium fuel system, to a thoughtful and rigorous attempt to replace the current system so as to make it much more robust and safety under DBA.

As of today, nitride fuels has been successfully tested in the sodium cooled BR-10 reactor in Russia [20] as well as in the FFTF and EBR-II test reactors [1]. Thus there are good grounds for considering nitrides as a strong candidate for Gen IV reactors due to their many advantages previously described and its results under operation. In this paper we will discuss different production routes as well as fuel recycling options for nitride fuels.

## Production

There are many different methods of producing nitrides with different pros and cons as will be outlined below. It is, however, clear that the use of different fissile nitrides will affect the production route considerably. If minor actinides are to be included their high specific radioactivity will more or less require a direct coupling to the separation solution to avoid unnecessary handling of fine, radioactive powder. However, if nitrides are to be based on mainly uranium and plutonium the challenges with respect to manufacture are similar as for normal mixed oxide fuel as has been shown in Russia where industrial nitride fuel manufacturing is ongoing [21].

### Production of nitrides from metal

The main advantage of using metallic uranium as starting material is that neither oxygen nor carbon need to be introduced at any stage of the process, hence virtually eliminating the issues of these elements appearing as impurities in the product, as is frequently the case with the carbothermic nitriding method. The different variants of the metal route are also carried out at lower temperatures, which reduces volatilisation in the manufacture of minor actinide-containing fuels. Additionally, and unlike in the carbothermic nitriding method where nitrogen is used in a double capacity as reactant and as carrier gas to remove formed carbon monoxide, quantitative incorporation of ^15^N can be achieved, which was first demonstrated with natural N_2_ [22] and later repeated with actual ^15^N_2_ [23]. Nitride production from metal has been demonstrated to be feasible in large scale [24, 25]. The main drawback is that high-purity uranium metal is required as feedstock. Metallic fuels have become uncommon in most contexts, there is almost no civilian production of enriched metallic uranium, and new industrial infrastructure would be required for large-scale nitride fuel production by this route. It should also be noted that all the powders involved, including the nitride itself, are more or less pyrophoric and can not be handled in the open. Densely sintered nitride pellets, on the other hand, are stable in air or water at ambient temperatures.

While there are a few technically different approaches, they are typically based on the sequential or combined reaction of uranium metal with hydrogen and nitrogen. The immediate product is, in the case of uranium, a more or less hyperstoichiometric sesquinitride which necessitates a concluding stoichiometry adjustment step consisting of denitriding in vacuum or under inert gas at 1100–1300 °C. Denitriding is not needed in the case of plutonium, which does not form a stable sesquinitride, but gives plutonium mononitride PuN as the immediate product from nitriding.

In principle, if not in practice, the reaction could be carried out just by exposing bulk uranium metal to nitrogen gas according to the reactions:

1 $$2{\text{U}} + \left( {\frac{3 + x}{2}} \right){\text{N}}_{2} \to {\text{U}}_{2} {\text{N}}_{3 + x}$$

2 $${\text{U}}_{2} {\text{N}}_{3 + x} \to 2{\text{UN}} + \frac{1 + x}{2}{\text{N}}_{2}$$

In actual production, several methods are employed to increase the reaction rate and yield, as outlined below.

#### Nitriding of metal with nitrogen gas

Solid uranium metal reacts sluggishly and often incompletely with elemental nitrogen. Even if the metal surface is thoroughly cleaned from oxide layers before the synthesis, initial surface nitriding of the metal produces a barrier which interferes with continued reaction [26]. One way of addressing this problem is to reduce the metal to a very fine UH_3_ powder by reaction with hydrogen gas [27] at 200–250 °C, and then reducing it back to metal at a temperature exceeding the dissociation temperature of the hydride but not causing melting or sintering of the metal powder [28]. Since the dissociation temperature is a function of the ambient partial pressure of hydrogen, vacuum or flowing argon is employed to keep this pressure low. The obtained metal powder, having a very large specific surface, will readily combine with N_2_. The reaction sequence thus becomes

3 $$2{\text{U}} + 3{\text{H}}_{2} \to 2{\text{UH}}_{3}$$

4 $$2{\text{UH}}_{3} \to 2{\text{U}} + 3{\text{H}}_{2}$$

followed by reactions (1) and (2).

One drawback, apart from the introduction of two additional steps, is that the uranium powder is in an exceptionally reactive state, will be very easily oxidised and has a tendency to self-ignition in air. Hence the nitriding step should be performed in immediate sequence and without any transfer of the powder. It is also noted [28] that the heat released in the exothermic nitriding reaction can cause caking or melting of the metal powder. A fluidised-bed setup is suitable in this as well as in several of the following variants of synthesis, as it both promotes fast and uniform reaction due to enhanced gas–solid interaction and reduces the risk for caking.

An alternative method is to melt uranium metal under nitrogen atmosphere, optionally at a pressure of several atmospheres. While small amounts of uranium nitride have been produced by arc melting [29], that method is not likely to be conveniently applied in industrial scale. In the case of conventional melting, such as in a crucible, surface nitriding still strongly interferes with the reaction between the liquid uranium and nitrogen gas [30].

#### Nitriding of hydride with nitrogen gas

Instead of reducing uranium hydride to form a reactive metal powder, the hydride can be reacted directly with N_2_. In addition to eliminating one process step, the endothermic character of the hydride decomposition reduces the overall reaction enthalpy and hence the risk for emergence of liquid uranium. In this method, hydriding is performed as above and the formed hydride is then nitrided with N_2_ at 300–500 °C [31]. The process will then start with reaction (3), followed by

5 $$2{\text{UH}}_{3} + \left( {\frac{3 + x}{2}} \right){\text{N}}_{2} \to {\text{U}}_{2} {\text{N}}_{3 + x} + 3{\text{H}}_{2}$$

and denitriding according to (2). After the stoichiometry adjustment step, the obtained powder is directly suitable for pressing and sintering.

**Fig. 2:**
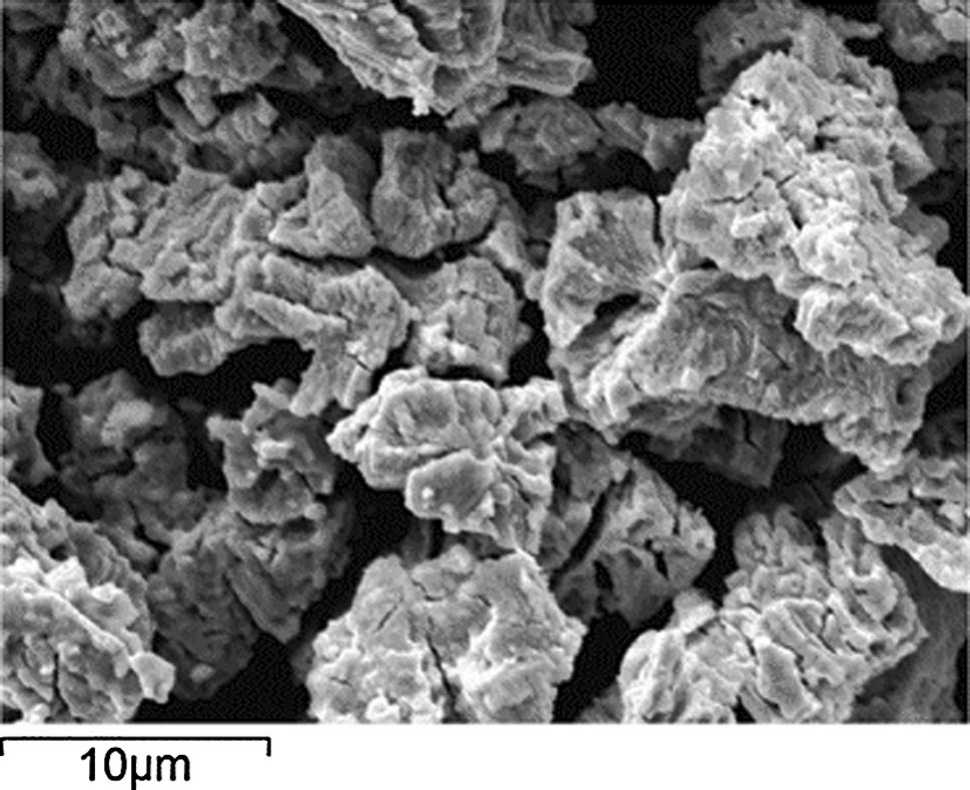
UN powder morphology image obtained by scanning electron microscopy [32]

The characteristic UN powder morphology, produced by hydriding–nitriding route with average particles size about 5 µm, is showed in Fig. 2 [32].

**Fig. 3:**
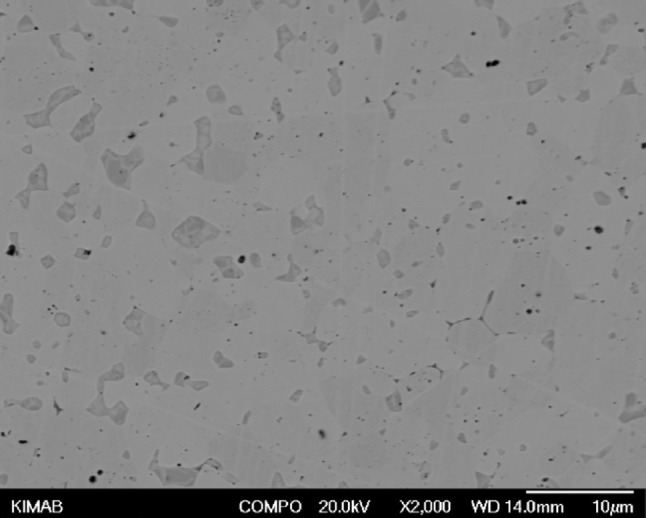
Scanning electron micrography of UN pellet sintered by SPS at 1650 °C and 3 min [31]

The as-synthesised UN powder, when sintering by spark plasma sintering (SPS) at 1650 °C and 3 min, forms a very high density (~ 99.8%TD) fuel with very low porosity, as presented in Fig. 3 [31].

#### Nitriding of metal with ammonia

An attractive feature of ammonia as nitriding agent is that the reaction can be carried out in a single step (except for the final stoichiometry adjustment). The reaction temperature, 200–300 °C, is even lower than for the nitriding of hydride, which would further lower any evaporation in the production of minor actinide-rich fuels for transmutation purposes.

The nitriding with ammonia involves two alternative paths [33]. The nitrogen in ammonia has higher chemical potential than that in N_2_, and hence direct nitriding of metal proceeds more readily and at lower temperature [34]. In a parallel reaction, uranium metal is hydrided, which may contribute to spalling and hence exposure of fresh metal surface, unlike in nitriding of metal with N_2_. In these respects, the method can be seen as a combination of reactions (1), (3) and (5) in parallel. However, the enhanced potential for direct nitriding of metal is related to the tendency of ammonia to partially decompose into N_2_ and H_2_ at the reaction temperature, and while this reaction will in the absence of catalysing surfaces be rather slow, nitriding works best if fresh ammonia is continuously supplied [33], and the N_2_ formed by NH_3_ decomposition will not be efficiently incorporated in the nitride at the low temperature used. Hence, if isotopically enriched ^15^N is used, the unconsumed fraction of NH_3_ and N_2_ will either represent a costly loss or have to be recycled.

**Fig. 4:**
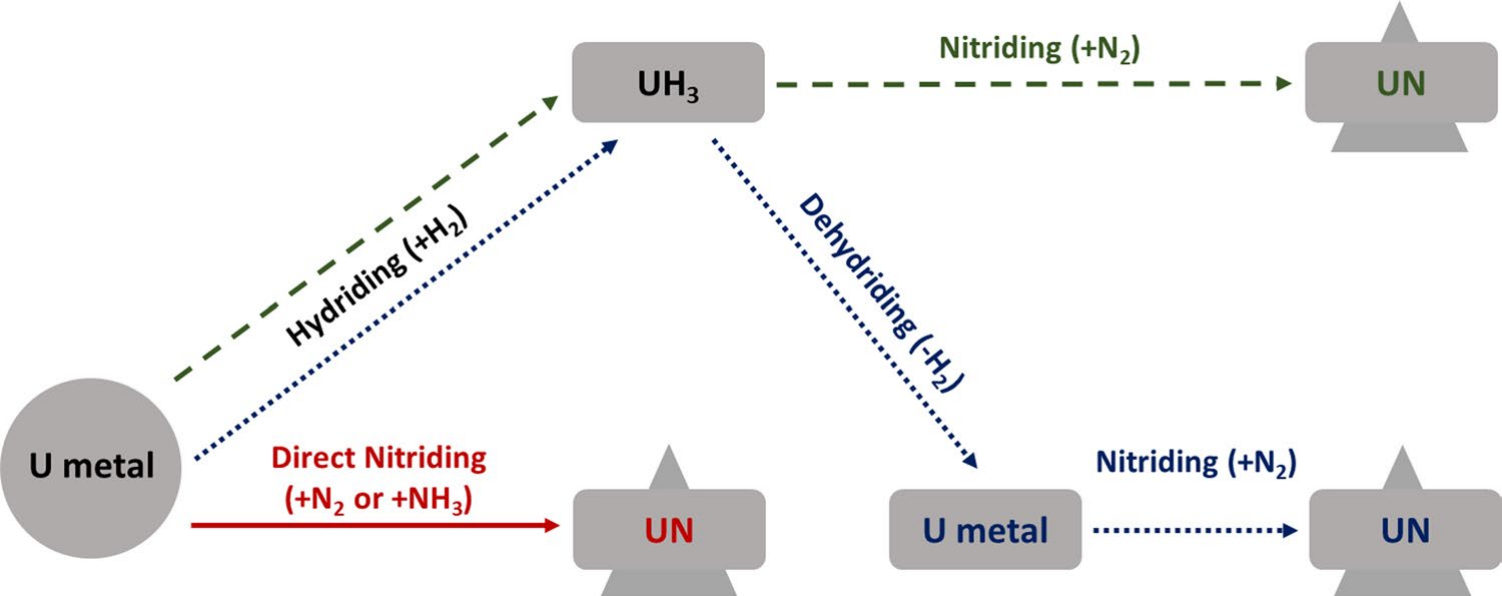
Overview of UN production from U metal: the solid line (red) represents the direct nitriding route; the dashed line (green) characterises the hydriding-nitriding route; and the dotted line (blue) illustrates the hydriding-dehydriding-nitriding route. (Color figure online)

To summarise the different uranium nitride routes from U metal, the following picture was made to illustrate all the above mentioned processes together (Fig. 4).

### Production of nitrides from fluorides

Even though synthesis by nitriding of uranium metal is in several ways both attractive and convenient, the round-about path over the metallic form is an undesirable complication from the point of industrial production. An obvious option would be to use uranium hexafluoride, UF_6_, or uranium tetrafluoride, UF_4_, which are industry-standard intermediaries in the isotopic enrichment process and in the production of uranium dioxide, thereby cutting out any preliminary conversion steps which not only increase cost and complexity, but also tend to introduce impurities. In the ammonolysis of uranium fluorides [35, 36], just as in the synthesis from metal, no carbon or oxygen compounds feature in the reaction, which allows a very high degree of purity of the product. Whether the starting material be UF_6_ or UF_4_ is not critical for the process, since the first step of reaction of ammonia with UF_6_ is reduction of the uranium to tetravalent shape, forming an adduct of UF_4_ and ammonium hydrogen fluoride NH_4_F [37]:

6 $$3{\text{UF}}_{6} + 8{\text{NH}}_{3} \to 3{\text{NH}}_{4} {\text{UF}}_{5} + 3{\text{NH}}_{4} {\text{F}} + {\text{N}}_{2}$$

The reaction is spontaneous at 100–200 °C, and the product can if so desired be decomposed to pure UF_4_ and NH_4_F by further heating to 500 °C [37], although this is unnecessary for the continued nitride synthesis.

From that point on, the reactions of UF_6_ or UF_4_ are essentially identical. Hence the choice between the fluorides will mostly depend on practical decisions concerning the process and equipment. The exclusive use of gaseous reactants (UF_6_ and NH_3_) to form a solid product can be an attractive feature when developing a continuous industrial process; on the other hand, UF_4_ has the advantage of being more easily handled. Also, since part of any ^15^NH_3_ used will form NH_4_F, and this amount is smaller with a less fluorinated raw material, the use of UF_4_ reduces the need of ammonia recycling.

The intermediate species formed in the nitriding reaction depend on the temperature and the composition of the gas phase, and involve tetravalent ammonium uranium fluoride complexes of the type (NH_4_)_*x*−4_UF_*x*_ nNH_3_, which formula can be rewritten as (UF_4_)(NH_4_F)_*x*_ nNH_3_. One suggested reaction formula [35] corresponds to

7 $$\left( {{\text{NH}}_{4} } \right)_{4} {\text{UF}}_{8} + 6{\text{NH}}_{3} \to {\text{UN}}_{2} + 8{\text{NH}}_{4} {\text{F}} + {\text{H}}_{2}$$

where associated ammonia molecules are omitted from the notation, and the product “UN_2_” is more correctly a nitrogen-rich form of uranium sesquinitride, which needs to be converted to mononitride in a final denitriding step according to (2).

### Production of nitrides from solution

Instead of working with fine powders during fuel production or simply connect the production directly to the separation/recycling process it is possible to substitute the powders with small actinide containing kernels commonly referred to as microspheres. The term “micro” should however be interpreted fairly freely since it is practically possible to produce spheres of various sizes and final sphere diameters typically ranges from around 50 µm up to about 1000 µm [38]. For a fully powder free production route, free flowing microspheres can be poured directly into cladding tubes. This concept is often referred to as sphere-pac fuel [39]. Alternatively the produced spheres can be compacted and sintered into traditional fuel pellets. This approach may at first glance look like a fully powder free process as well but any final physical homogenization of the sintered pellets by grinding and polishing will introduce a step where a limited fraction of powder is generated.

There are several different gelation techniques available for the production of microspheres. Common for all techniques is that the starting material is an aqueous metal solution that is somehow dispersed as droplets and solidified into kernels. The solidification process can generally be divided into two sub-types. In the first type a precipitation is induced in the droplets by extraction of water and/or acid from the droplets into the dispersion media. The second type is based on gelation by pH increase in the droplets causing the metal ions to gel/precipitate. The results presented here are based on the second type of gelation using a method called the internal gelation process.

#### The internal gelation process

The internal gelation process was originally not developed for the production of nitride based materials but rather for the production of uranium oxide fuel kernels [40, 41]. Through continues research and development over the years the process has been adapted to production of oxides, nitrides and carbides and work has been performed on both single metal containing microspheres as well as metal mixes. Microspheres containing among others U, Zr, Pu and mixed U–Pu, Zr–Ce and Zr–Pu have been produced by various internal gelation processes [42–47].

The internal gelation process is based on temperature induced pH increase causing the metal ions in the sol to gel/precipitate. Generally the method starts with a metal nitrate solution. The solution is generally cooled down to in between 0 and 4 °C [38]. Urea and Hexamethylenetetramine (HMTA) are added to the sol, either as solids to dissolve or as a mixed solution. The HMTA is the principal gelation agent in the sol while urea is added as a complexation agent in order to prevent premature gelation of the sol. If the aim is to produce carbide or nitride a carbon containing source, commonly a fine carbon powder, is also dispersed in the sol. The sol is introduced as small droplets into an immiscible heat carrier commonly heated to in between 50 and 100 °C [42, 48]. When the droplets are heated in the immiscible heat carrier the HMTA degrades, this causes an increase in pH in the droplets which results in gelation/precipitation of the metal ions in the droplet.

More work has been carried out on production of uranium based microspheres using the internal gelation process as compared to gelation of trans-uranium actinides or gelation of inert matrix materials such as Zr. The principal reaction steps of the gelation process are therefore presented using gelation of uranium as the model system. The principal steps pf the internal gelation of uranium based sols have been determined to [49].

- Decomplexation of urea:
  8 $${\text{UO}}_{2} \left[ {{\text{CO}}\left( {{\text{NH}}_{2} } \right)_{2} } \right]_{2}^{2 + } \rightleftharpoons 2{\text{CO}}\left( {{\text{NH}}_{2} } \right)_{2} + {\text{UO}}_{2}^{2 + }$$
- Hydrolysis of the uranyl ions:
  9 $${\text{UO}}_{2}^{2 + } + 2{\text{H}}_{2} {\text{O}} \rightleftharpoons {\text{UO}}_{2} \left( {\text{OH}} \right)_{2} + 2{\text{H}}^{ + }$$
- Protonation of HMTA:
  10 $$\left( {{\text{CH}}_{2} } \right)_{6} {\text{N}}_{4} + {\text{H}}^{ + } \rightleftharpoons \left[ {\left( {{\text{CH}}_{2} } \right)_{6} {\text{N}}_{4} {\text{H}}} \right]^{ + }$$
- HMTA decomposition:
  11 $$\left[ {\left( {{\text{CH}}_{2} } \right)_{6} {\text{N}}_{4} {\text{H}}} \right]^{ + } + 3{\text{H}}^{ + } + 4{\text{NO}}_{3}^{ - } + 6{\text{H}}_{2} {\text{O}} \rightleftharpoons 4{\text{NH}}_{4}^{ + } + 4{\text{NO}}_{3}^{ - } + 6{\text{CH}}_{2} {\text{O}}$$

The continuous hydrolysis of the uranyl ions that lead to the formation of a solid material is therefore driven forward by removal of H^+^ from the system by reaction with the HMTA.

After gelation of the spheres has been completed they are washed in order to remove silicone oil and excess gelation chemicals. First the silicone oil is washed of using for example petroleum ether or carbon tetrachloride. After the silicone oil has been washed of the spheres are generally washed/aged in aqueous ammonia. The reason for washing/ageing in aqueous ammonia solution is twofold. The ageing part is purposed to supply an abundance of hydroxyl ions for the microspheres to make sure that all the uranyl ions have been properly gelled/precipitated. The washing of the microspheres in aqueous solution is performed in order to remove ions, such as NH_4_^+^ and NO_3_^−^ for example, as well as residual gelation chemicals from the formed microspheres. Residual gelation chemicals and NH_4_NO_3_ in the dry spheres will increase the degree of mechanical damage in the spheres during heat treatments, such as the carbothermic reduction, when the residual chemicals are decomposed to gases and leaves the microspheres.

**Fig. 5:**
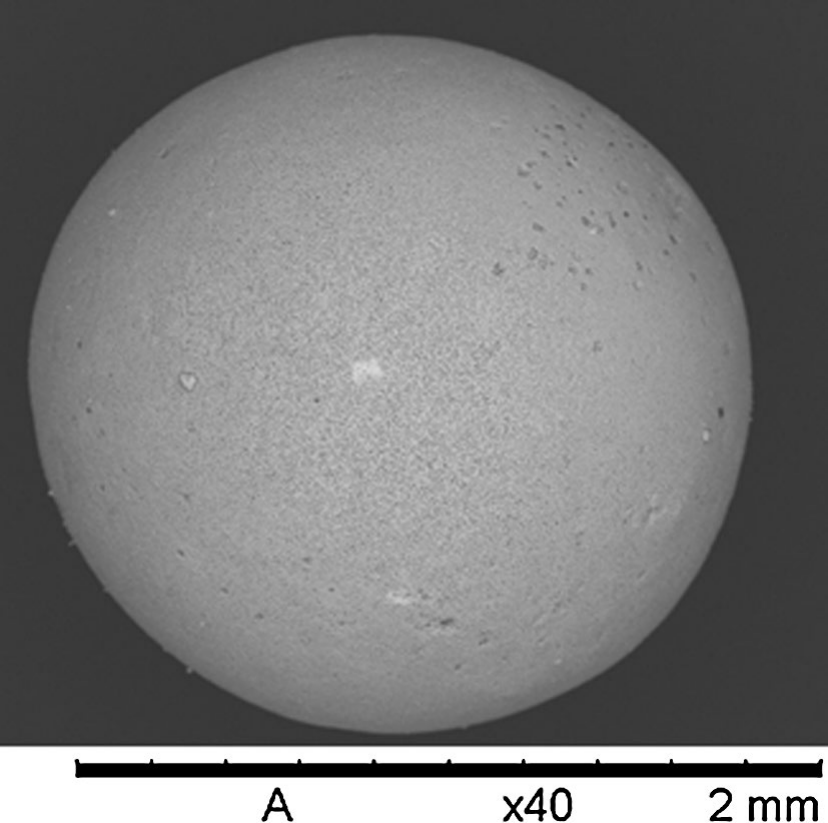
SEM image showing an example of a carbon containing uranium microsphere after washing and drying. The microsphere in the image contain a carbon to metal molar ration of 2.15 [50]

The microspheres produced by the internal gelation technique are generally smooth and of fairly good spherical shape. Examples of washed and dried microspheres prior to any heat treatment are presented in Fig. 5 [50].

Choosing a suitable carbon source is detrimental to the success in making the final nitride material. Since the conversion of metal oxide microspheres to metal nitride involves the solid state reaction between carbon and metal oxide, and no milling of the microspheres can be performed if the goal is to have a low dust process, it is important that the carbon is well dispersed in the microspheres already at the gelation stage to provide beneficial conditions for the nitride formation.

**Fig. 6:**
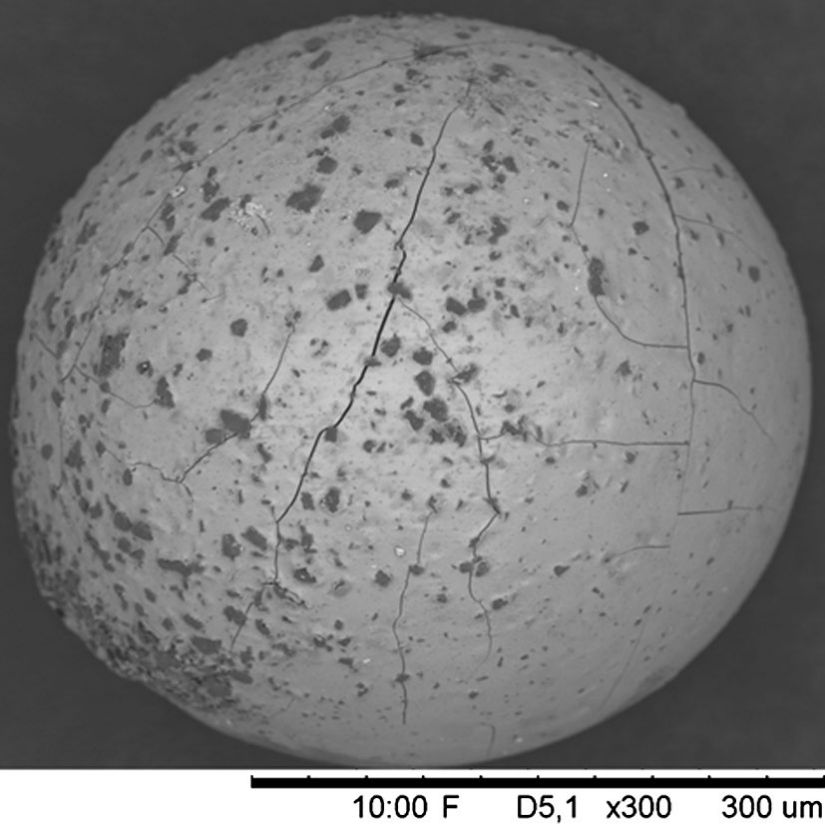
SEM image illustrating a washed and dried zirconium based microsphere using a course graphite powder as carbon source. The molar carbon to metal ratio in the microsphere is 2.3 [50]

The effect of gelation performed using a carbon source with low dispersion and short settling time during the gelation process is showed in Fig. 6 [50].

**Fig. 7:**
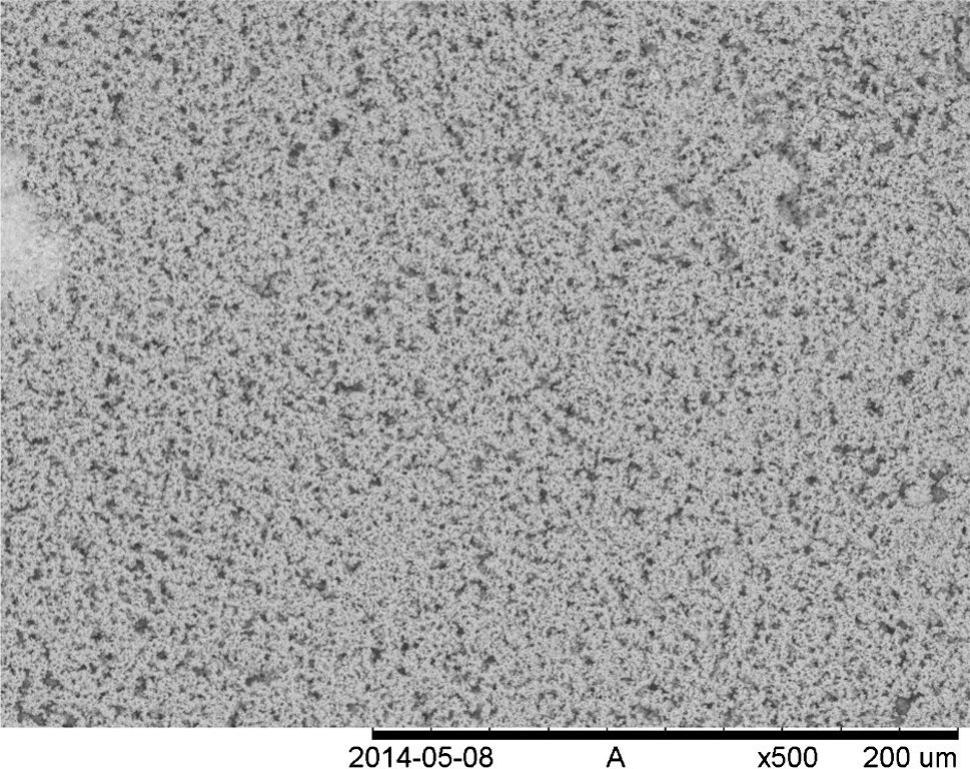
Larger magnification image of the surface of the microsphere in Fig. 5. As can be seen also at large magnification there is no clear aggregation of carbon particles observable on the surface of the microsphere [50]

From Figs. 5 and 6 the comparison becomes skewed since Fig. 5 is taken at much lower magnification but also when comparing to larger magnification images (Fig. 7) it can be seen that the microspheres produced in the batch represented in Fig. 5 has much more homogeneous carbon distribution [50].

Both microspheres in Figs. 5 and 6 have been prepared using graphite powder as carbon source but the main difference is that the particle size of the carbon used in Fig. 5 is a bit below 0.5 µm while the carbon particles in Fig. 6 have a large size fraction ranging up to about 10–20 µm size.

In addition to the difference in carbon distribution within the microspheres it is also observable in Figs. 5 and 6 that the microspheres in the images are of very different size. When producing materials using the internal gelation process it is possible to control the size of the microspheres by controlling the gelation parameters used during production. The size of the droplets that are introduced into the heat carrying silicone oil can be controlled by the size of the nozzle that the sol is dripped through and by physically breaking up the sol into small droplets at the nozzle tip by application of oscillation of the nozzle. It is also possible to affect the size of the dried microspheres by changing the metal concentration in the sol. As the metal concentration in the sol decreases the microsphere will shrink more during drying and the final size of the dried microspheres will decrease.

#### Carbothermic reduction

Carbothermic reduction is a nitride production technique that utilizes metal oxides as starting material. The name derives from that carbon is mixed into the metal oxide material as a reduction agent in order to facilitate the removal of oxygen from the system during the reaction. If the dioxide of the metal to be nitrided is used as input material in the synthesis, such as UO_2_ or PuO_2_ for example, a minimum carbon to metal molar ratio of 2 is required to theoretically eliminate all oxygen from the sample and produce a nitride material without oxygen impurities. However, in reality the reaction systems experiences limitations due to the inability to produce infinitely fine mixtures of carbon and metal oxide. In order to produce nitride materials low in oxygen impurities using reasonable reaction times, carbon is commonly added in excess to the reaction system compared to the stoichiometric requirements. This means that in practice, carbon to metal ratios exceeding the theoretical molar ratio 2 are used in order to suppress residual oxygen impurities in the final nitride materials. Carbothermic reduction can be utilized for nitride production from all of the actinide oxides, ranging from uranium to curium, which are the elements usually of interest when discussing production, implementation and recycling of nuclear fuels in a Generation IV fuel cycle. It is however not the case that a single specific set of reaction parameters is the perfect optimum regardless of which one of the actinide oxides one wishes to synthesize. On the contrary, the reaction conditions and optimal carbon to metal ratio for carbothermic reduction varies depending on if it is U, Np, Pu, Am or Cm oxide that is being converted into nitride [51–54].

Production of actinide nitrides by carbothermic reduction has been performed on all of the above mentioned elements, either in pure form or in actinide mixes, across the world. The majority of synthesis experience however derive from production of uranium nitride, which is why uranium will be used as model element when describing the different reactions involved in the synthesis.

When producing uranium nitride the first step is to remove excess oxygen present in the uranium oxide either as uranium trioxide, UO_3_, or as hyper stoichiometric uranium dioxide, UO_2+*x*_. This can be achieved by reduction using hydrogen in inert carrier gas as reducing agent [55]

12 $${\text{UO}}_{2 + x} + x{\text{H}}_{2} \rightleftharpoons {\text{UO}}_{2.0} + x{\text{H}}_{2} {\text{O}}$$

If carbon is already present in the system, as in the case of when gelation derived microspheres are being nitride for example, some carbon in the system can be lost as carbon monoxide due to oxidation by steam [56]

13 $${\text{C}} + {\text{H}}_{2} {\text{O}}\left( {\text{g}} \right) \rightleftharpoons {\text{CO}} + {\text{H}}_{2}$$

An alternative to using hydrogen is to use carbon as reduction agent [47, 57]

14 $${\text{UO}}_{3} + 0.5{\text{C}} \rightleftharpoons {\text{UO}}_{2} + 0.5{\text{CO}}_{2}$$

Note that reactions (12) and (13) are model reactions illustrating one case when H_2_ is used as reducing agent and one case when carbon is used as reducing agent. One could equally well have balanced reaction (12) with UO_3_ on the left reaction side and reaction (14) with UO_2+*x*_ on the left reaction side.

When the actinide oxide has been brought to the desired oxidation state the carbothermic reduction reaction can be carried out. The nitrogen required for the nitride formation during the carbothermic reduction reaction is supplied via the reaction atmosphere. Different reaction atmospheres such as pure N_2_, mixed N_2_ + H_2_ or NH_3_ can be used [57–60].

When the carbothermic reduction is performed in N_2_ as reaction atmosphere and the temperature is kept below 1723 K it has been reported to proceed by [57]:

15 $${\text{UO}}_{2} + 2{\text{C}} + 0.5{\text{N}}_{2} \rightleftharpoons {\text{UN}} + 2{\text{CO}}$$

Above 1723 K a carbonitride is reported to form instead according to [57]:

16 $${\text{UO}}_{2} + \left( {2 + x} \right){\text{C}} + \left[ {\left( {1{-}x} \right) /2} \right]{\text{N}}_{2} \rightleftharpoons {\text{UN}}_{1 - x} {\text{C}}_{x} + 2{\text{CO}}$$

The conformation of carbide in the system during carbothermic reduction can theoretically be removed by prolonged heat treatment by

17 $${\text{UN}}_{1 - x} {\text{C}}_{x} + \left( {x /2} \right){\text{N}}_{2} \rightleftharpoons {\text{UN}} + x{\text{C}}$$

Elimination of carbide from the nitride material is thus depending on the ability to remove elemental carbon from the reaction mixture. As long as there is a free carbon phase to equilibrate against in the system the final nitride material will contain carbide impurities. It has been estimated that as long as an equilibrating carbon phase exists in the system the UN material produced will contain about 15 mol% carbide [61]. One possible removal mechanism is the reaction between excess carbon and unreacted UO_2_ according to reaction (15). However, since carbon to metal ratios higher than the theoretical 2 needs to be applied in order to efficiently produce nitrides with low oxygen impurity levels there will obviously not be enough UO_2_ present in the system to remove all elemental carbon as CO. Therefore some additional mechanism for carbon removal needs to be present in order to drive reaction (17) forward.

One possibility to remove carbon is by addition of H_2_ to the reaction atmosphere. By introducing H_2_ into the N_2_ atmosphere the elemental carbon can be converted into hydrogen cyanide according to the reaction [62]:

18 $${\text{H}}_{2} + {\text{N}}_{2} + 2{\text{C}} \rightleftharpoons 2{\text{HCN}}$$

Limitations due to chemical equilibrium in reaction (18) can be overcome by using flowing reaction gas, thus purging the system of formed HCN and allowing carbon to be eliminated from the solid sample. Thermodynamic modelling made on non-uranium containing nitride fuel has also identified HCN as the likely reaction product during decarburization of the nitride material during synthesis [63].

An advantage of using the carbothermic reduction process compared to other processes for nitride production is that it has been identified as a process that may be more well suited for scaling up to large scale production compared to other nitride production processes [64].

**Fig. 8:**
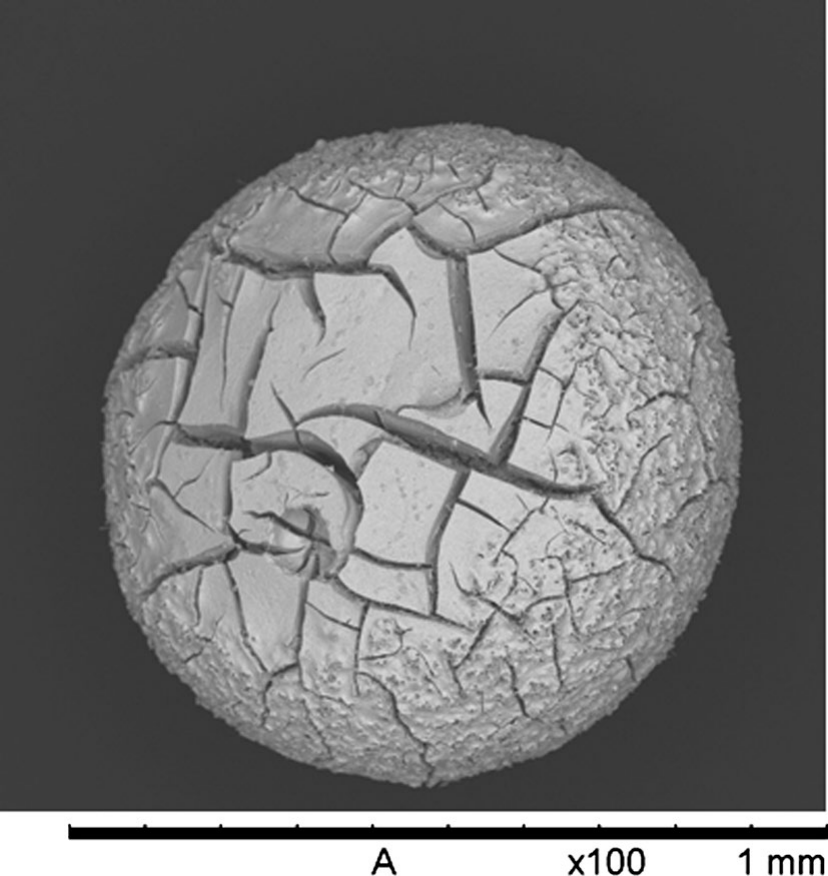
SEM image of a ZrN microsphere after carbothermic reduction. The sphere has fractured severely during the heat treatment step followed by partial sintering during the late stages of the carbothermic reduction. This results in a damaged final microsphere containing zones of partly sintered ZrN [50]

**Fig. 9:**
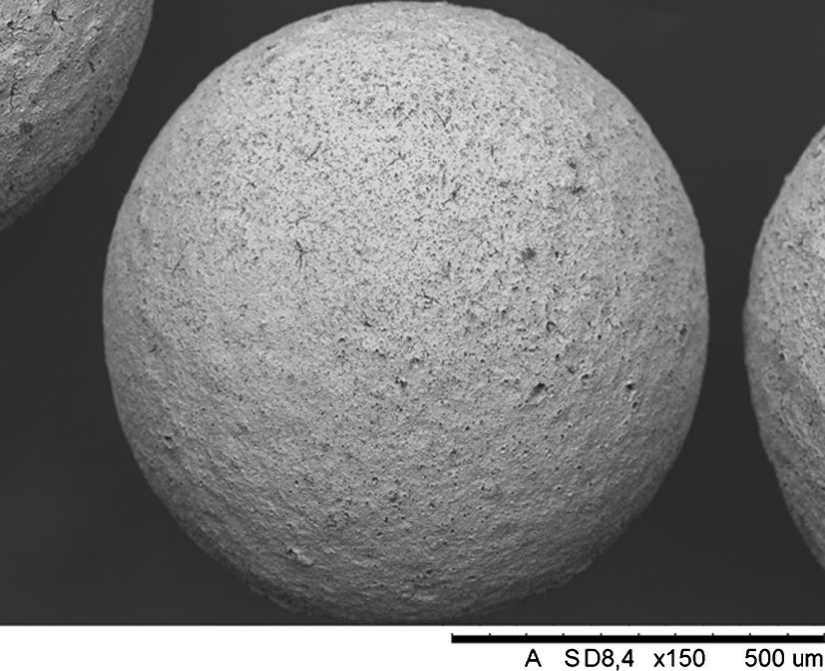
Example of a SEM image of a UN microsphere after carbothermic reduction. The sphere is covered in small pores but essentially the sphere has survived the heat treatment steps without suffering any physical damage [50]

The resulting microspheres, after carbothermic reduction has been performed, can possess a wide range of microstructures. Depending on how much of the residual chemicals that was washed out of the microspheres post gelation and what heating ramps and reaction temperatures that are being used during the carbothermic reduction the final structure of the microspheres can be strongly affected. Examples of possible nitride microsphere structures are presented in Figs. 8 and 9 [50].

In every microsphere batch produced there will of course be internal variance with some microspheres showing higher or lower degree of physical damage but on average the structure of the final microspheres can be controlled by controlling the heat treatment of the materials. The main difference between Figs. 8 and 9, apart from the different metal in the microspheres, is the heating rate. The ZrN microsphere example in Fig. 8 was heated at 20 °C/min up to a maximum temperature of 1700 °C. The UN microsphere in Fig. 9 was heated at a rate of 3 °C/min to 350 °C followed by 10 °C/min to 800 °C to remove residual chemicals and moisture before being cooled down. The oxide + carbon spheres formed was subsequently heated 15 °C/min to a maximum temperature of 1650 °C during the carbothermic reduction itself. This is an illustration of how the microsphere microstructure can be controlled in order to potentially produce materials with tailored suitability for use either pellet pressing or directly as sphere-pac fuel.

Reaching high nitride purity using the carbothermic reduction process is not straight forward and is dependent on metal oxide to be nitrided, the heat treatment applied during carbothermic reduction, on the carbon to metal ratio used and on the degree of homogenous mixing of the carbon with the metal oxide. Measurement of impurities can be performed either by direct measurement of the oxygen, carbon and nitrogen levels in the final nitride but also by indirect measurements such as X-ray diffraction (XRD). X-ray diffraction of the materials can, apart from detecting undesired phases in the material such as residual oxides, also estimate solid solution impurities by comparing the lattice parameter of the produced nitride to reference literature data. Shifts in lattice parameter can be used to estimate for example carbide impurities in nitride by making an interpolation between the lattice parameters of metal nitride and carbide according to Vegards law. Examples of nitride purities that can be achieved using carbothermic reduction are presented in Table 1. The data in Table 1 is a selected compilation of data from [45], PuN-data, [65], two first ZrN-data points and [66], mixed ZrN-PuN data and the remainder being hitherto unpublished data. The table presents purities measured either by direct measurement of N, O, and C content and C content estimated by Vegards law from XRD data.

**Table 1:** Examples of nitride purities reachable by the carbothermic reduction technique when producing metal nitrides of Zr, U and Pu

Desired material	Nitrogen content (wt%)	Oxygen content (wt%)	Carbon content (wt%)	Carbon content (wt%) according to XRD
UN	5.43	0.29	0.011	0.08
UN	5.31	0.10	0.005	0.13
UN	5.26	0.08	0.212	0.36
UN	4.03	0.24	2.27	1.84
PuN	–	–	–	0.17
PuN	–	–	–	0.14
PuN	–	–	–	0.31
PuN	–	–	–	0.06
(Zr0.6Pu0.4)N	–	–	–	0.95
ZrN	–	–	6.11	5.53
ZrN	–	–	2.06	2.98
ZrN	10.50	0.36	2.96	2.12

The (–) notation in the table indicates not measured

None of the high activity samples in Table 1 was measured for direct determination of N, O or C content, the reason being radiation safety based at the facility where the work was performed. However in all the samples in Table 1 where C-content is estimated only from XRD data no residual oxide phase could be detected and the impurity estimate is therefore made on solid solution carbonitride materials. The impurities presented in those materials are thus carbon impurities in otherwise fairly pure nitride materials.

#### Pellet fabrication from nitride microspheres

Nitrides used for nuclear fuel applications are generally difficult materials to sinter compared to when making oxide fuel. Due to high melting temperatures nitride fuels need to be sintered at high temperatures [67]. The tendency of the fuel nitrides, such as UN or PuN for example, to dissociate into metal an nitrogen at high temperatures and low N_2_ partial pressures adds to the challenge of successfully sinter these materials [68]. Pressing pellets from nitride microspheres adds an additional challenge to the task. Since the nitrides are hard materials mechanical compaction of the microspheres into good quality green pellets becomes increasingly harder as the porosity of the microspheres decreases. The level of residual porosity and degree of fracturing in the microspheres is in turn dependent on the thermal treatment applied when converting the gelled spheres into nitrides. In order to produce a microsphere based material suitable for mechanical compaction it is thus important to factor in the final mechanical properties of the spheres when designing the heat treatment steps and not only the design criteria with respect to final nitride purity.

**Fig. 10:**
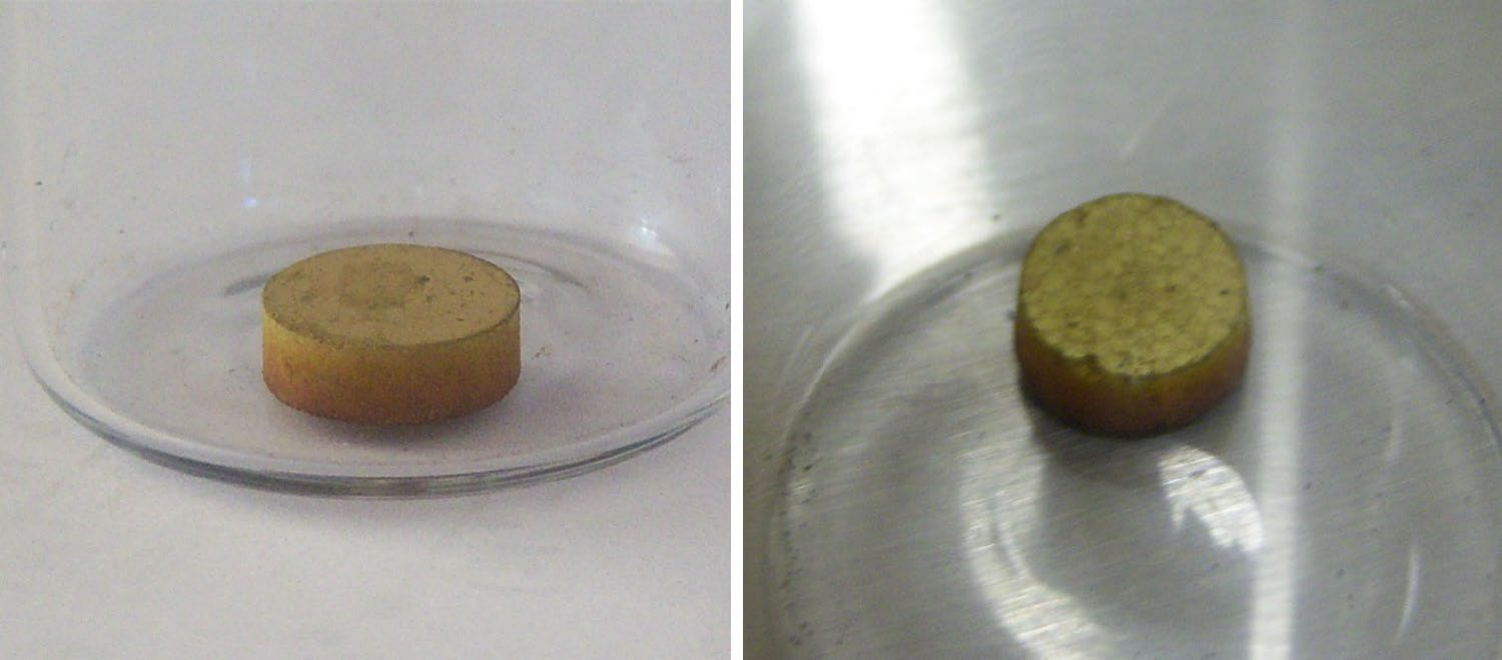
Comparison between two ZrN pellets sintered at 2000 °C. There is a distinct difference observable by ocular inspection between the pellets based on if the microspheres pressed were destroyed or retained their individual shape during compaction [50]

An example of the behavior of final ZrN pellets in which one of the pellets was made from microspheres that were largely disintegrated during compaction compared to when the microspheres retained their individual shape are shown in Fig. 10. Both pellets are pressed using the same compaction pressure and sintered at 2000 °C in flowing N_2_ gas [50].

An alternative to conventional pressure less sintering of the nitride microspheres would be to apply some version of field assisted sintering instead. One potential technique could be spark plasma sintering (SPS). SPS has been successfully applied to sinter UN powders to pellets reaching densities close to the theoretical densities [69]. Application of SPS to sinter nitride microspheres has been performed to sinter ZrN [68]. Densities of final ZrN pellets could be increased using SPS compared to pressure less sintering. In that study ZrN pellet a maximum density of about 87% of theoretical density was achieved when sintering ZrN microspheres at 1700 °C for 30 min in argon using a pressure of 99 MPa during sintering. No study of sintering of UN microspheres by SPS technique exist to this date to the authors’ knowledge.

## Dissolution and separation

In order to close the nuclear fuel loop it is vital that after use the used fuel can be dissolved and then the useful components can be separated out for re-manufacture. In the case of nitride fuels the main challenge is the dissolution step.

Uranium nitride can in itself be dissolved in nitric acid (HNO_3_) much in the same way as conventional UO_2_ fuels [70]. This has been further demonstrated with pure as well as with carbon- and oxygen-rich UN pellets [71]. Hence reprocessing by the PUREX method appears to be feasible. The practical experience with irradiated nitride fuels is however rather limited. A large quantity of spent (~ 80 GWd/ton) (U,Pu)N fuel was successfully reprocessed by a somewhat modified acid dissolution and liquid extraction process, in which dissolution was assisted by a small addition of fluoride [72]. In particular, actinide nitride pellets with a large admixture of zirconium nitride have been shown to be exceptionally difficult to dissolve without addition of fluoride ions or hydrofluoric acid HF (which too become equivalent in the nitric acid environment) [71]. Considering that Zr is one of the most abundant fission products, it can not be excluded that it can interfere with the nitric acid dissolution of high-burnup, pure UN fuels unless HF is added to the solution.

If the fuel is enriched in ^15^N, the nitrogen must for economical reasons be recycled. Dissolution in HNO_3_ produces nitrogen-containing gases by several reactions, with part of this nitrogen coming from the acid and hence being of natural isotopic composition [73]. As some of these gaseous species will form from both the nitride and the acid, the result is an isotopic dilution which would necessitate costly re-enrichment of ^15^N. It will therefore be necessary to recover the ^15^N before the dissolution in nitric acid, most likely by a preceding step of voloxidation. This could be done by combustion of the fuel in oxygen, forming NO_2_, to be further converted to a suitable reagent for re-use, or oxidising it in superheated steam [74], forming NH_3_ which is directly suitable for manufacture of new fuel. It has however been noted that a significant fraction of nitrogen remains in the oxidic product even after rather extensive oxidation [11], and complete oxidation to UO_3_, which can not be achieved with steam, is likely needed to liberate all the ^15^N.

If on the other hand natural nitrogen (> 99% ^14^N) is used in the fuel manufacture, the additional issue (besides the neutron economy penalty) of ^14^C production must be dealt with. The feasibility of ^14^C capture has been demonstrated [72], and ^14^C is already separated and stored in industrial-scale reprocessing [75]. Yet this results in an additional long-lived waste fraction that can be avoided if ^15^N is used.

Once the nitride fuel has either been directly dissolved (ignoring the ^15^N issue) or following an oxidative dissolution the resulting solutions can be used directly in one of the newly developed Grouped Actinide Extraction Systems (GANEX) [76–78]. These processes were developed to have no pure stream of e.g. plutonium so all actinides are extracted together (except uranium) thus making the processes considerably more proliferation safe and at the same time potentially opening the possibility to a direct connection to the fuel fabrication line using a wet process.

Electrometallurgical dissolution and refining (often called pyroprocessing) is an often suggested alternative to aqueous reprocessing methods, and has the advantage that the ^15^N component can be recovered in undiluted form. Such methods of electrolytical dissolution into a molten salt medium appear to be particularly suitable for nitrides because of their appreciable electric conductivity. This approach has been shown to work for unirradiated (U,Pu)N fuel [79]. An overview of different technical solutions can be found in [80]

## Conclusions

The development of nitride fuels has been going on and off in the last 5 decades. From the beginning they were promising as nuclear fuels due to their high fissile density and thermal conductivity. However, unfavourable reactions with hot water as well as a more complicated production made them obsolete compared oxides in the 60ies. Today the nitrides got a new spring. Both for use as accident tolerant fuels as well as fuel in fast, metal cooled reactors for Gen IV systems. Research is ongoing on how to ease the production and to overcome the interactions with water. Industrial scale production has begun in e.g. Russia.
